# Twelve-Month Prevalence and Changes in Driving After Drinking: United States, 1991–1992 and 2001–2002

**Published:** 2006

**Authors:** S. Patricia Chou, Bridget F. Grant, Deborah A. Dawson, Frederick S. Stinson, Tulshi Saha, Roger P. Pickering

**Affiliations:** Laboratory of Epidemiology and Biometry, Division of Intramural Clinical and Biological Research, National Institute on Alcohol Abuse and Alcoholism, National Institutes of Health, Department of Health and Human Services, Bethesda, MD

**Keywords:** Alcohol-impaired driving, Drinking and driving, Sociodemographic characteristics, High risk subgroups, Changes in drinking and driving

## Abstract

**Background:**

Drinking and driving has been identified as one of the most important contributors of motor vehicle fatalities. This paper addressed the existing gap in our public health knowledge regarding the current prevalence of driving after drinking and how this has changed over the past decade.

**Methods:**

Prevalence rates of drinking and driving in 2001–2002, and changes in those prevalence rates between 1991–1992 and 2001–2002, were examined in two large nationally representative surveys of the U.S. population.

**Results:**

Overall, the prevalence of driving after drinking was 2.9 percent in 2001–2002, representing approximately six million U.S. adults. This rate was about three-quarters of the rate observed in 1991–1992 (3.7 percent), reflecting a 22-percent reduction. Generally, the male–female differentials in the rate of driving after drinking decreased over the past decade. However, the sex ratios increased substantially for underaged youth over the past decade, reflecting the sharp decrease in prevalence of driving after drinking among 18- to 20-year-old women. Constant and emerging subgroups at high risk for drinking and driving included Whites, Native Americans, males, underaged young adults, and 21- to 25-year-olds.

**Conclusions:**

The results of this study highlighted the need to continue to monitor prevalence and changes in driving after drinking. Results are discussed in the context of strengthening existing prevention and intervention efforts and developing new programs with the sociodemographic differentials observed in this study in mind.

## Introduction

Road traffic injuries and fatalities are a global problem affecting all sectors of society. The World Health Organization (WHO) predicted that 1.39 million people would die in road traffic crashes in 2000 and that 2.34 million people will die in 2020 (Murray and Lopez, 1996). Among them, more than 50 percent of traffic fatalities occurred among young to middle-aged adults ages 15–44. In 2000, road traffic crashes ranked as the ninth leading cause of mortality and morbidity, accounting for 2.8 percent of all global deaths and disability. WHO’s projections suggest that by 2020, road traffic injuries could rank third among all causes of death and disability worldwide (WHO, 2003).

The practice of drinking and driving has been consistently identified as one of the strongest predictors of motor vehicle fatalities worldwide (WHO, 2003). In the United States, approximately 40 percent of all motor vehicle–related deaths are attributed to alcohol each year (National Highway Traffic Safety Administration [NHTSA], 1995). In the year 2001 alone there were over 16,000 alcohol-related fatalities and 275,000 alcohol-related injuries as a result of motor vehicle crashes (NHTSA, 2002). Accordingly, the consequences of drinking and driving have received wide media coverage and intense attention from public policy makers, and the practice of drinking–driving has been studied using a variety of data sources.

The National Highway Traffic Safety Administration (NHTSA) maintains the Fatal Accident Reporting System (FARS), which collects detailed information for every fatal traffic crash occurring in the United States. Yearly data are released for public use by the following year and have been used to identify demographic and drinking characteristics associated with a high risk of fatal injuries (e.g., Kennedy et al., 1996). FARS data have also been studied in conjunction with blood alcohol concentration (BAC) data collected in a series of national roadside surveys. Using such an approach, Lund and Wolfe (1988, 1991) and Zador (1991) reported that each 0.02-percent increase in the driver’s non-zero BAC nearly doubled the risk of being involved in a fatal crash. In addition, the 1973, 1986, and 1996 national roadside surveys indicated the prevalence of drinking drivers (BAC ≥ 0.05 percent) fell sharply from 1973 (13.7 percent) to 1986 (8.4 percent) but decreased slightly between 1986 and 1996 (7.7 percent). Voas and colleagues (1998) further reported that there was no reduction in the prevalence of drivers at the highest BACs between 1973 and 1996 despite the significant decline in the overall prevalence of drinking drivers on American roadways.

The Behavioral Risk Factor Surveillance System (BRFSS) is a nationwide annual telephone survey of U.S. adults that has also collected data on drinking–driving in addition to other health-related behaviors. Smith and Remington (1989), using data from the 1986 BRFSS, estimated that there were 150 million drinking–driving events in the United States in 1986. Based on data from the 1993 BRFSS, Liu and colleagues (1997) estimated there were 123 million episodes of alcohol-impaired driving among U.S. adult drivers, a decrease of about 20 percent compared with the 1986 estimate. This rate of reduction was comparable with the 30-percent decline in alcohol-related traffic fatalities during the period of 1982 and 1992 (Centers for Disease Control and Prevention, 1993).

As evidence indicates that automobile fatalities are the leading cause of death among American youth 15–20 years old (Centers for Disease Control and Prevention, 1996), numerous studies have focused specifically on young drivers. Using the FARS data, a special report on young drivers compiled by NHTSA (2002) reported that the number of young drivers (15–20 years old) who were intoxicated at the time of the fatal crash dropped 24 percent between 1991 and 2001. In addition, prior studies have analyzed data collected in national surveys to investigate drinking-and-driving behaviors among youth. For example, data collected in the 1991 Youth Risk Behavior Survey (Escobedo et al., 1995) indicated that the prevalence of drinking and driving was positively associated with frequency of alcohol use and binge drinking and years elapsed since the initiation of alcohol use, and data from the 1999 National Household Survey on Drug Abuse (NHSDA) indicated that college attendance increased the likelihood of drinking–driving regardless of any pre-existing conditions (Paschall, 2002). Likewise, data from the National Institute on Alcohol Abuse and Alcoholism’s (NIAAA) 1991–1992 National Longitudinal Alcohol Epidemiologic Survey (NLAES) and the 1999 Harvard College Alcohol Survey showed that drinking–driving was inversely related to age at first drink and at first intoxication (Hingson et al., 2002, 2003). None of these studies based on national survey data examined changes over time in drinking and driving among youth. However, based on drinking driver fatalities reported in the FARS data, NIAAA surveillance reports (NIAAA, 1993, 2003) showed a 15-percent decline in alcohol-related fatal traffic crashes among youth ages 16–24 between the years 1991 and 2001 (31.4 percent versus 26.9 percent).

From a review of previous research, it became apparent that few studies provided prevalence information on alcohol-impaired driving at the national level or within important subgroups of the population. Nor did many of the studies track changes in the prevalence of alcohol-impaired driving over time within major subgroups of the population. From a public health policy standpoint, it is essential to be able to assess the prevalence of driving after drinking too much, along with changes of the prevalence over time both at the national level and within major subgroups of the population. In light of the bold national goal established in 1995 to reduce alcohol-related automobile crash fatalities to 11,000 by 2005 (NHTSA, 1997), the progress in achieving this goal has been slow, and the change that occurred in the past few years has been in the wrong direction (Sweedler et al., 2004). Accurate and timely information will help formulate effective and empirically derived prevention and intervention programs. This is of critical importance for those highly vulnerable subgroups of the population, namely college students and young adult males. However, available data sources to address these concerns are surprisingly sparse.

The purpose of this paper, therefore, was to address the existing gap in the public health knowledge regarding the 12-month (current) prevalence and changes in drinking and driving over the past decade among important subgroups of the U.S. population. To address changes in drinking-and-driving practices, we compared data from two large nationally representative samples of the U.S. population: the 1991–1992 National Longitudinal Alcohol Epidemiologic Survey (NLAES, *n* = 42,862) and the 2001–2002 National Epidemiologic Survey on Alcohol and Related Conditions (NESARC, *n* = 43,093).

## Methods

### Samples

Both the 1991–1992 NLAES and the 2001–2002 NESARC are nationally representative samples of the general adult population (age 18 and older) of the United States conducted by the National Institute on Alcohol Abuse and Alcoholism. Details of these two surveys have been described in detail elsewhere (Grant et al., 1992, 2003). Face-to-face interviews were conducted with each respondent drawn from the civilian, noninstitutionalized population of the United States for both NLAES and NESARC. NESARC additionally included a subsample of respondents residing in group quarters. Overall survey response rates were 90 percent for NLAES and 81 percent for NESARC.

To ensure adequate numbers of respondents for analytic purposes, over-sampling of Blacks in NLAES and of Blacks and Hispanics in NESARC was implemented at the design phase. Within each household unit, a person age 18 or older was randomly selected. Young adults (ages 18–29 in NLAES and ages 18–24 in NESARC) were oversampled at a rate of 2.25:1.00 at this stage of sample selection to secure a greater representation of this heavier drinking subgroup of the population. Both samples were weighted to adjust for oversampling young adults and nonresponse at the household and person levels. The data were then adjusted to be representative of the U.S. population for a variety of socioeconomic variables including region, sex, race, and ethnicity using the 2000 Decennial Census.

### Measure of Driving After Drinking

On NESARC, driving after drinking was explored by asking “In your entire life, did you EVER more than once drive a car, motorcycle, truck, boat, or other vehicle after having too much to drink?” A followup question, “Did that happen in the past 12 months?” was asked if the answer to the preceding question was “yes.” There was a slight variation in the question on the NLAES survey, in which the question read, “In the last 12 months, did you drive a car, motorcycle, truck, boat, or other vehicle after having too much to drink?” A followup question—“About how many times did this happen in the last 12 months?”—was asked if the answer to the preceding question was “yes.” To equate these measures, only NLAES respondents reporting driving after having too much to drink more than once were included in the analyses presented here.

### Analysis Procedures

To account for the complex sampling design of both NLAES and NESARC, a specialized software program, SUDAAN (Research Triangle Institute, 2004), was used to estimate the standard errors of all current prevalence estimates. This software adopts Taylor series linearization to take into account the design effects of complex sample surveys like NLAES and NESARC. Differences in prevalence rates of driving after drinking for the total sample and among important sociodemographic subgroups of the population were conducted using *t*tests designed for independent samples.

## Results

### Twelve-Month Prevalence of Driving After Drinking: 2001–2002

The 12-month prevalence rates of driving after drinking by age, sex, and race are presented in Table 1. In the total sample, the 12-month prevalence of driving after drinking was 2.9 percent, representing approximately six million American adults. The prevalence of driving after drinking among males was 4.4 percent, whereas the corresponding rate among females was 1.5 percent, representing 4.4 million and 1.6 million U.S. adults, respectively.

#### Age

Young adults ages 18–29 (5.3 percent) were significantly more likely to drive after drinking in the last 12 months, compared with 30- to 44-year-olds (3.5 percent) and 45- to 64-year-olds (1.9 percent), with age differentials of 1.5 and 2.8, respectively. Respondents age 65 or older had the lowest prevalence of operating a motor vehicle after drinking (0.2 percent, *p* < 0.001). When examined within the race–ethnicity and sex groups, the prevalence of driving after drinking was significantly (*p* < 0.001) greater among young adults (ages 18–29) compared with the rates of all other age groups, but only among White males.

#### Race–Ethnicity

Native Americans exhibited the highest rate of driving after drinking (4.1 percent), which was significantly (*p* < 0.05) greater than those of Blacks (1.5 percent), Asians (1.4 percent), and Hispanics (2.1 percent). Notably, the rates of the latter three ethnic groups did not significantly differ. When prevalence was examined with respect to gender, significantly greater rates were observed among Native Americans relative to Whites, Blacks, Asians, and Hispanics, and this difference was statistically significant among males and females.

#### Gender

Overall, in the last 12 months, males were significantly more likely to operate a motor vehicle after drinking too much than females (4.4 percent versus 1.5 percent), a ratio of about 2:93. Among race–ethnic groups, however, we observed no gender difference among Native Americans and Asians. In contrast, males showed significantly greater rates than females among the other ethnic groups (i.e., Whites, Blacks, and Hispanics). The sex ratios varied from 2:9 to 3:7 for these three ethnic groups.

Gender differences were observed within age–ethnicity subgroups of the population. In general, males were significantly more likely to drive after drinking compared with females for most age groups among Whites, Blacks, and Hispanics. Due to several prevalence rates not meeting precision standards, we were not able to assess the gender differences among the age–ethnic subgroups of Native Americans and Asians.

#### 18- to 29-Year-Olds

To address the elevated prevalence of driving after drinking among the young adult group (18- to 29-year-olds) we disaggregated this age category into single ages and present the prevalences and associated population estimates by sex in Table 2. This detailed table showed that among male and female adults, the prevalence of driving after drinking peaked at ages 22 and 23. With respect to gender differentials in the prevalence of 12-month driving after drinking, male rates were significantly greater than female rates in all age groups except 21- and 23-year-olds.

### Changes in Prevalence of Driving After Drinking Between 1991–1992 and 2001–2002

In the total sample, the prevalence of driving after drinking significantly declined 22 percent over the past decade, from 3.7 percent to 2.9 percent (Table 3). Significant declines in driving after drinking were also observed among males (5.8 percent versus 4.4 percent) and Whites (4.1 percent versus 3.3 percent). There were no significant changes in the rate of driving after drinking observed among females. With regard to age groups in the total sample, the only significant decreases in the rates of driving after drinking were among 18- to 29-year-olds overall and among 18- to 29-year-old Whites.

Among males, significant decreases in the rates of driving after drinking were observed among Whites overall (6.4 percent versus 5.0 percent), Hispanics overall (5.4 percent versus 3.3 percent), and among 18- to 29-year-old males (11.6 percent versus 7.8 percent), 18- to 29-year-old Whites (13.9 percent versus 9.9 percent), and 18- to 29-year-old Hispanics (8.8 percent versus 5.0 percent).

The picture was different for females. Significant reductions in the rates of driving after drinking were noted for 18- to 29-year-old females overall (4.2 percent versus 2.9 percent) and 30- to 44-year-old Black females (1.5 percent versus 0.4 percent) over the last decade. In contrast, small but significant increases in the corresponding rates were observed among females ages 45–64 (0.4 percent versus 0.9 percent) and among 18- to 29-year-old Hispanic females (0.6 percent versus 2.0 percent).

To further examine changes in driving after drinking among 18- to 29-year-olds, we disaggregated age into 1-year intervals for that age group (Table 4). In the total sample, significant declines in the prevalence of driving after drinking were only observed among 18-, 26-, 27-, 28-, and 29-year-olds. Among males, the rate of driving after drinking significantly decreased only among 21-, 26-, 27-, and 29-year-olds.

## Discussion

This paper examined the prevalence of driving after drinking, which is identified as one of the major contributors to automobile crash risk and subsequent injury and premature death. A variety of deterrence-based initiatives have been introduced and implemented during the past decade. A significant and concurrent reduction has been observed in rates of drinking–driving and alcohol-related automobile crashes in most developed countries. In 1982, there were 26,173 alcohol-related fatalities in the United States. By 2002, alcohol-related fatalities fell to 17,419, representing a 33.4-percent reduction (Sweedler et al., 2004). Our results indicated that the overall 12-month prevalence of drinking–driving was 2.9 percent in 2001–2002, representing approximately six million U.S. adults. This rate was approximately three-quarters of the rate (3.7 percent) observed in 1991–1992, representing a 22-percent reduction. This translated into a 13-percent reduction in alcohol-involved traffic fatalities between 1991 and 2001 (NHTSA, 2002) and is consistent with findings from the three national roadside surveys and FARS, which clearly document reductions in drinking–driving occurrences and in highway injuries and fatalities over the past three decades (Voas et al., 1998). The reduction rate of 22 percent in driving after drinking reported in our study measured about half of the overall decline as noted between 1982 and 2002. Our results also indicated that among young adults (18- to 29-year-olds), there was a 33-percent decline in the prevalence of drinking–driving over the past decade (from 7.9 percent in 1991–1992 to 5.3 percent in 2001–2002). Based on analysis of drinking driver fatalities of the FARS data, NIAAA surveillance reports (NIAAA, 1993, 2003) showed a 15-percent decline in alcohol-related fatal traffic crashes among youth ages 16–24 between 1991 and 2001 (31.4 percent versus 26.9 percent).

The decrease in driving after drinking varied across major sociodemographic subgroups of the population. Overall, there was a decrease in the gender difference in prevalence of driving after drinking over the last decade (the sex ratio decreased from 3:41 to 2:93). This male–female convergence was partly due to a greater reduction in prevalence among White and Hispanic males than among their female counterparts. Furthermore, White middle-aged females (ages 45–64) and Hispanic young adult females showed significant increases in prevalence over the past decade, causing the gender gap to diminish even further. Echoing this narrowing gender gap, detailed analyses on drivers using the FARS data over the same time period revealed that among all alcohol-related fatal crashes, the male-to-female ratio in alcohol-related fatal crashes dropped from 9 in 1991 to about 5.4 in 2001 (NIAAA, 1993, 2003).

This convergence in male and female prevalence rates was further examined with respect to individual ages among the young adult age group, where our detailed analysis showed contradictory results. Most significantly, sex ratios in fact increased substantially for under-aged youth (ages 18–20) over the past decade, reflecting the sharp decrease in prevalence among 18- to 20-year-old women. This finding demonstrates how aggregating age groups can have a significant impact on study results and their implications. This is especially true with respect to drinking and driving, because crash risks are highest at the extremes of the age spectrum (Williams, 2003). It is not clear why most age groups showed a convergent gender gap while underage youth did not. Perhaps over the past decade, a greater number of the underage females worked or lived off campus, or drove a motor vehicle under the “designated driver” program because they were less impaired than their male friends. Future research examining the risk of drinking–driving among college students as a function of living arrangement (e.g., on-campus versus off-campus) is warranted.

Despite long-standing prevention and intervention efforts, our results showed that drinking–driving remained prevalent among young adult males (7.8 percent) in 2001–2002 even though the rate had decreased since 1991–1992 (11.6 percent). Our detailed analysis further showed that about 1 in 10 young adult males ages 22 and 23 (prevalence rates of 11.5 and 10.4, respectively) posed significant risk on the roadways because of their hazardous driving practices. The vulnerability of young adults is consistent with previous findings that being young and being male are strong demographic correlates of drinking and driving (Wilson and Jonah, 1985; Gruenewald et al., 1996). It is also consistent with common perceptions of vulnerability of young adult males who are prone to risk-taking behaviors such as driving after drinking. An alternative explanation for this phenomenon might be the link between drinking and driving and specific drinking contexts. For example, young adult males might have a tendency to drink at friends’ homes or in public places such as bars, lounges, or restaurants—contexts that all increase the probability of driving after drinking.

Among young adult males between 18 and 29 years old, White youth were at the greatest risk (9.9 percent) for driving after drinking. This rate was substantially greater than those of their Black, Asian, and Hispanic counterparts, consistent with earlier studies showing that the prevalence of drinking and driving among young minority groups is lower than that of Whites (Ross et al., 1991). In the search for explanations for this observed gender and race–ethnic differential, future research should take into account numerous environmental factors, including social and economic conditions as mediators of the rate changes. Among certain minority subgroups of the population, historical factors, cultural beliefs and practices, and stress and tension induced by racial discrimination need to be considered in planning and designing effective prevention and intervention programs.

In any research when data regarding a socially unacceptable behavior such as driving after drinking are assessed, the prevalence tends to be underestimated. With respect to this “social undesirability bias,” it has been suggested that these self-reported measures gathered with well-designed surveys can, nonetheless, be useful, even essential tools, for assessing individual-level epidemiological relationships (Greenfield and Rogers, 1999; Babor and Del Boca, 1992). Thus, our study’s data on sociodemographic correlates of driving after drinking and changes in prevalence over the past decade, derived from this large nationally representative data set with oversampling of important high-risk groups such as young adults, offer valuable insights for planning and designing intervention and prevention programs to address this important public health challenge.

In summary, the availability of the NLAES and NESARC data obtained 10 years apart allows constant and emerging high-risk subgroups of the population with respect to driving and drinking to be identified with much greater clarity and specificity than in the past. Whites, Native Americans, males, and 18- to 29-year-olds demonstrated the highest rates of driving and drinking in 1991–1992, rates that have remained consistently high in 2001–2002, despite decreases in rates during that time period. Emerging risk subgroups of the population include 45- to 64-year-olds and 18- to 29-year-old Hispanic females, who demonstrated small but significant increases in the rate of driving after drinking over the past decade. However, our detailed age analyses for young adults identified most underage young adults (ages 18–20) and young adults ages 21–25 at high risk of driving after drinking, as evidenced by the stability in rates among these younger age groups over the past 10 years. Taken together, these results highlight the need to strengthen existing prevention and intervention efforts and to develop new programs designed with these sociodemographic differentials in mind.

Most countries worldwide experienced declines in automobile crashes and traffic fatalities in the early 1980s and 1990s. The progress in reducing mortality from road traffic injury in many countries has since stalled or even reversed (Sweedler et al., 2004). Despite steady declines in alcohol-related traffic fatalities in the United States between 1982 and 1994 (Sweedler et al., 2004), the trend seems to have reversed in the past 3 years, during which the mortality rates due to sustained traffic injuries increased slightly (Hingson et al., 2003). This reversing trend, viewed within the context of billions of dollars of direct or indirect costs due to traffic crashes each year (Blincoe and Faigne, 1992; NHTSA, 2002), continues to cause great public health concern in contemporary America. When the data of Wave 2 NESARC become available, we will examine a host of risk factors that might differentiate individuals who continue to drive after drinking from those who mature out of such a dangerous drinking practice. These analyses promise to inform future prevention and intervention programs beyond the contributions to such efforts reflected in this study.

## Figures and Tables

**Table 1:** Twelve-Month Prevalence of Driving After Drinking by Age, Sex, and Race: United States, 2001–2002

Age (years)	Male			Female			Total		
		
	%	S.E.	Population Estimate^a^	%	S.E.	Population Estimate^a^	%	S.E.	Population Estimate^a^
Total									
Total	4.4	0.22	4388	1.5**	0.11	1585	2.9	0.13	5973
18–29	7.8	0.57	1759	2.9**	0.30	659	5.3	0.33	2418
30–44	5.2	0.36	1653	1.8**	0.20	598	3.5	0.23	2251
45–64	3.0	0.31	929	0.9**	0.16	301	1.9	0.18	1231
≥ 65	0.3	0.10	47	—	—	26	0.2	0.07	73
White									
Total	5.0	0.25	3516	1.7**	0.13	1296	3.3	0.15	4812
18–29	9.9	0.76	1364	3.5**	0.38	492	6.7	0.42	1857
30–44	6.1	0.45	1301	2.5**	0.28	542	4.2	0.29	1842
45–64	3.4	0.38	810	1.0**	0.19	251	2.2	0.22	1061
≥ 65	0.3	0.12	41	—	—	11	0.2	0.06	52
Black									
Total	2.6	0.35	258	0.7**	0.17	90	1.5	0.19	348
18–29	3.1	0.74	84	1.2*	0.45	40	2.1	0.42	124
30–44	3.5	0.74	118	0.4**	0.15	18	1.8	0.33	136
45–64	1.8	0.43	54	—	—	32	1.3	0.30	85
≥ 65 b	—	—	2	0.0	0.00	0	0.1	0.05	2
Native American									
Total	5.9	1.61	124	2.4	0.92	56	4.1	0.84	180
18–29	—	—	32	—	—	18	5.8	2.24	50
30–44	9.9	3.25	69	—	—	11	5.5	1.70	80
45–64	—	—	19	—	—	12	2.0	0.50	31
≥ 65	—	—	4	—	—	15	—	—	19
Asian									
Total	2.0	0.61	89	0.8	0.34	35	1.4	0.33	124
18–29	4.3	1.63	57	2.6	1.29	32	3.5	1.00	89
30–44	—	—	24	—	—	4	—	—	27
45–64	—	—	8	0.0	0.00	0	—	—	8
≥ 65	0.0	0.00	0	0.0	0.00	0	0.0	0.00	0
Hispanic/Latino									
Total	3.3	0.48	402	0.9**	0.18	107	2.1	0.27	509
18–29	5.0	1.00	222	2.0**	0.50	76	3.6	0.60	298
30–44	3.1	0.59	141	0.6**	0.16	24	1.9	0.32	165
45–64	1.5	0.42	39	—	—	7	0.9	0.22	45
≥ 65	0.0	0.00	0	0.0	0.00	0	0.0	0.00	0

a Population estimates are in thousands.

b Estimate does not meet precision standard.

* *p* < 0.05 for gender comparison.

** *p* < 0.01 for gender comparison.

**Table 2:** Twelve-Month Prevalence of Driving After Drinking by Sex and Age: United States, 2001–2002

Age (years)	Male			Female			Total		
		
	%	S.E.	Population Estimate^a^	%	S.E.	Population Estimate^a^	%	S.E.	Population Estimate^a^
Total									
18	5.5	1.43	122	1.0**	0.58	20	3.4	0.80	142
19	6.4	1.50	140	2.8*	0.94	50	4.8	0.91	191
20	9.3	2.34	175	1.8**	0.72	39	5.3	1.17	214
21	7.2	1.60	135	5.7	1.67	119	6.4	1.18	254
22	11.5	2.12	199	4.2**	1.31	77	7.8	1.26	276
23	10.4	2.16	183	7.1	1.48	124	8.7	1.29	307
24	6.9	1.49	127	2.8*	0.84	54	4.8	0.83	182
25	9.4	2.62	179	2.3*	0.84	44	5.9	1.40	223
26	6.9	1.96	119	1.4**	0.47	26	4.1	1.01	145
27	7.5	1.92	138	2.4*	0.86	43	5.0	1.08	181
28	7.1	1.69	121	2.0**	0.69	38	4.4	0.86	159
29	6.3	1.68	121	1.3**	0.52	24	3.9	0.92	145
30–44	5.2	0.36	1653	1.8**	0.20	598	3.5	0.23	2251
45–64	3.0	0.31	929	0.9**	0.16	302	1.9	0.18	1231
≥ 65	0.3	0.10	47	— b	—	26	0.2	0.07	728

a Population estimates are in thousands.

b Estimate does not meet precision standard.

* *p* < 0.05 for gender comparison.

** *p* < 0.01 for gender comparison.

**Table 3:** Changes in the 12-Month Prevalence of Driving After Drinking by Age, Sex, and Race: 1991–1992 and 2001–2002

Race–ethnicity/age (years)	Male				Female				Total			
		
	NLAES (1991–1992)		NESARC (2001–2002)		NLAES (1991–1992)		NESARC (2001–2002)		NLAES (1991–1992)		NESARC (2001–2002)	

	%	S.E.	%	S.E.	%	S.E.	%	S.E.	%	S.E.	%	S.E.
Total												
Total	5.8	0.22	4.4**	0.22	1.7	0.11	1.5	0.11	3.7	0.13	2.9**	0.13
18–29	11.6	0.53	7.8**	0.57	4.2	0.34	2.9*	0.30	7.9	0.33	5.3**	0.33
30–44	5.8	0.34	5.2	0.36	1.8	0.18	1.8	0.20	3.8	0.20	3.5	0.23
45–64	3.0	0.33	3.0	0.31	0.4	0.08	0.9**	0.16	1.6	0.16	1.9	0.18
≥ 65	0.3	0.11	0.3	0.10	— a	—	—	—	—	—	0.2	0.07
White												
Total	6.4	0.26	5.0**	0.25	2.0	0.12	1.7	0.13	4.1	0.15	3.3**	0.15
18–29	13.9	0.67	9.9**	0.76	5.5	0.42	3.5**	0.38	9.8	0.42	6.7**	0.42
30–44	6.7	0.41	6.1	0.45	2.0	0.21	2.5	0.28	4.3	0.24	4.2	0.29
45–64	3.1	0.36	3.4	0.38	0.4	0.10	1.0**	0.19	1.7	0.18	2.2	0.22
≥ 65	0.3	0.13	0.3	0.12	—	—	—	—	—	—	0.2	0.06
Black												
Total	2.9	0.46	2.6	0.35	0.6	0.16	0.7	0.17	1.7	0.23	1.5	0.19
18–29	4.7	1.07	3.1	0.74	0.4	0.20	1.2	0.45	2.4	0.54	2.1	0.42
30–44	2.8	0.69	3.5	0.74	1.5	0.44	0.4*	0.15	2.1	0.40	1.8	0.33
45–64	1.9	0.73	1.8	0.43	—	—	—	—	0.9	0.33	1.3	0.30
≥ 65	—	—	—	—	0.0	0.00	0.0	0.00	—	—	—	—
Native American												
Total	4.2	2.05	5.9	1.61	5.8	2.40	2.4	0.92	5.1	1.49	4.1	0.84
18–29	—	—	—	—	13.2	5.46	—	—	9.2	3.15	5.8	2.24
30–44	—	—	9.9	3.25	—	—	—	—	—	—	5.5	1.70
45–64	—	—	—	—	0.0	0.00	—	—	—	—	2.1	0.50
≥ 65	0.0	0.00	—	—	0.0	0.00	—	—	0.0	0.00	—	—
Asian												
Total	1.1	0.51	2.0	0.61	1.2	0.77	0.7	0.34	1.2	0.46	1.4	0.33
18–29	2.3	1.39	4.3	1.63	—	—	2.6	1.29	2.8	1.33	3.5	1.00
30–44	—	—	—	—	—	—	—	—	—	—	—	—
45–64	0.0	0.00	—	—	0.0	0.00	0.0	0.00	0.0	0.00	—	—
≥ 65	0.0	0.00	0.0	0.00	0.0	0.00	0.0	0.00	0.0	0.00	0.0	0.00
Hispanic												
Total	5.4	0.79	3.3*	0.48	0.6	0.19	0.9	0.18	3.0	0.41	2.1	0.27
18–29	8.8	1.49	5.0*	1.00	0.6	0.25	2.0*	0.50	4.7	0.77	3.6	0.60
30–44	3.6	0.99	3.1	0.59	1.2	0.48	0.6	0.16	2.4	0.57	1.9	0.32
45–64	—	—	1.5	0.42	—	—	—	—	2.1	1.00	0.9	0.22
≥ 65	0.0	0.00	0.0	0.00	0.0	0.00	0.0	0.00	0.0	0.00	0.0	0.00

a Estimate does not meet precision standard.

* *p* < 0.05.

** *p* < 0.01.

**Table 4:** Changes in the 12-Month Prevalence of Driving After Drinking by Sex and Age: 1991–1992 and 2001–2002

Age (years)	Male				Female				Total			
		
	NLAES (1991–1992)		NESARC (2001–2002)		NLAES (1991–1992)		NESARC (2001–2002)		NLAES (1991–1992)		NESARC (2001–2002)	

	%	S.E.	%	S.E.	%	S.E.	%	S.E.	%	S.E.	%	S.E.
Total	5.8	0.22	4.4**	0.22	1.7	0.11	1.5	0.11	3.7	0.13	2.9**	0.13
18	8.3	2.00	5.5	1.43	4.1	1.30	1.0*	0.58	6.2	1.17	3.4*	0.80
19	7.5	1.75	6.4	1.50	5.8	1.90	2.8	0.94	6.7	1.28	4.8	0.91
20	10.3	1.80	9.3	2.34	5.0	1.24	1.8*	0.72	7.9	1.10	5.3	1.17
21	14.0	2.48	7.2*	1.60	3.1	0.87	5.7	1.67	8.0	1.24	6.4	1.18
22	15.6	2.25	11.5	2.12	6.0	1.35	4.2	1.31	10.8	1.38	7.8	1.26
23	12.6	2.05	10.4	2.16	5.4	1.20	7.1	1.48	9.2	1.25	8.7	1.29
24	10.1	1.60	6.9	1.48	2.9	0.78	2.8	0.84	6.5	0.91	4.8	0.83
25	10.6	1.82	9.4	2.62	3.4	0.96	2.3	0.84	6.7	1.00	5.9	1.40
26	14.4	1.91	6.9**	1.96	3.7	0.90	1.4*	0.47	9.2	1.08	4.1**	1.02
27	12.6	1.70	7.5*	1.92	4.1	0.93	2.4	0.86	8.4	0.97	5.0*	1.08
28	10.6	1.64	7.1	1.69	3.6	0.77	2.0	0.69	7.0	0.87	4.4*	0.86
29	12.8	1.74	6.3**	1.68	4.0	0.82	1.3**	0.52	8.5	1.00	3.9**	0.92
30–44	5.8	0.34	5.2	0.36	1.8	0.18	1.8	0.20	3.8	0.20	3.5	0.23
45–64	3.0	0.33	3.0	0.31	0.4	0.08	0.9**	0.16	1.6	0.16	1.9	0.18
≥ 65	0.3	0.11	0.3	0.10	— a	—	—	—	—	—	0.2	0.07

a Estimate does not meet precision standard.

* *p* < 0.05.

** *p* < 0.01.
